# The fibrolytic potentials of vitamin D and thymoquinone remedial therapies: insights from liver fibrosis established by CCl4 in rats

**DOI:** 10.1186/s12967-016-1040-4

**Published:** 2016-09-29

**Authors:** Abdelghany Hassan Abdelghany, Mohammad A. BaSalamah, Shakir Idris, Jawwad Ahmad, Bassem Refaat

**Affiliations:** 1 Department of Anatomy, Faculty of Medicine, Alexandria University, Alexandria, Egypt; 2 Laboratory Medicine Department, Faculty of Applied Medical Sciences, Umm Al-Qura University, Al Abdeyah, PO Box 7607, Makkah, Saudi Arabia; 3 Pathology Department, Faculty of Medicine, Umm Al-Qura University, Al Abdeyah, Makkah, Saudi Arabia

**Keywords:** Vitamin D, Thymoquinone, Fibrogenesis, Fibrolytic, Immunomodulatory, Carbon tetrachloride

## Abstract

**Background:**

Vitamin D (VitD) and thymoquinone (TQ) are nutraceutical agents with well-known immunomodulatory and hepatoprotective properties. This study measured whether VitD and TQ, individually or combined, could have direct fibrolytic activities and/or enhanced performance during remedial treatment of liver fibrosis established by CCl4 in rats.

**Methods:**

Eighty five male Wistar rats were used as 10 negative controls (NC) and the remainders were distributed equally into 5 groups: short (S-PC) and long (L-PC) positive controls, TQ, VitD and VitD/TQ groups. CCl4 was injected for 7 weeks followed by a week of no intervention. TQ and/or VitD were given orally (3 days/week) from week 9 and euthanasia was at week 17 for all groups except the S-PC was at week 9. Following histopathological and digital image analyses, TGF-β1, IL-6, IL-10, IL-22 and MMP-9 were measured by ELISA in liver homogenates while the corresponding cytokine receptors were measured by immunohistochemistry. The mRNA expressions of all molecules were measured by quantitative RT-PCR.

**Results:**

Fibrosis was evident in both PC-groups and was significantly more advanced in the L-PC than S-PC, reaching to cirrhosis. The concentrations of TGF-β1, IL-6, IL-22 and their receptors were significantly higher (P < 0.05) simultaneously with significantly lower (P < 0.05) concentrations of MMP-9, IL-10 and IL-10 receptors in the S-PC and L-PC than the NC-group. TQ and VitD monotherapies showed significantly less fibrosis than L-PC but were similar to S-PC. Both remedial monotherapies also resulted in significant decreases of TGF-β1, IL-6, IL-22 and their receptors together with significant increases of MMP-9 and IL-10 system compared with S-PC and L-PC groups. Interestingly, dual therapy resulted in the most significant improvement in fibrosis score and index, yet was significantly higher (P < 0.05) than the NC-group, and concurred with the utmost significant restorations of all candidate genes and proteins.

**Conclusions:**

VitD and TQ exhibited comparable anti-fibrogenic effects and modulated several pro- and anti-fibrotic mediators. Additionally, VitD/TQ dual therapy alleviated the previously established liver fibrosis simultaneously with significantly enhanced actions at the molecular level. More studies are required to explorer the therapeutic value of TQ and VitD against liver fibrosis in human.

**Electronic supplementary material:**

The online version of this article (doi:10.1186/s12967-016-1040-4) contains supplementary material, which is available to authorized users.

## Background

Hepatic fibrosis and later the development of cirrhosis are major health concerns causing 1.5 million annual deaths worldwide [1]. Liver fibrosis is a wound-healing response to chronic liver inflammation that ultimately results in the progressive accumulation of extracellular matrix (ECM) and distortion of normal liver architecture [2]. Chronic liver inflammation may result from several well-known risk factors such as viral infection, drug use, autoimmune hepatitis, alcohol abuse and metabolic disorders [3]. Hepatic stellate cells (HSCs) are the main cell type to produce ECM in the liver and, following sustained liver injury, they trans-differentiate from a quiescent to an active state with the formation of fibrotic scar tissue [1–3]. Activated HSCs mediate abnormal degradation of the main components of ECM such as type IV collagen and increase the deposition of collagen type I and III with over-expression of the cytoskeleton protein; α-smooth muscle actin, resulting in an excess of non-functioning ECM [2].

At the molecular level, several cytokines are involved in the regulation of immune responses to liver injury as well as activation of HSCs [3, 4]. Transforming growth factor (TGF)-β1 and interleukin (IL)-6 are pro-fibrotic cytokines that are upregulated during chronic liver inflammation and their serum and tissue concentrations correlate positively with the degree of liver fibrosis [5, 6]. On the other hand, IL-10 exhibits anti-fibrotic activities and an increase in the hepatic expression of the cytokine and its receptors (IL10RA & IL10RB) have been associated with significantly less scar formation following a variety of liver injuries [7, 8]. IL-22 is another cytokine produced mainly by T-helper (Th)-17, Th-22 and natural killer cells which are enriched in the intrahepatic environment [9]. This cytokine is known to play a critical role in liver immunity and its receptor (IL-22R) is expressed by several liver cells including HSCs [9–13]. However, the role of IL-22 in the liver whether as inducer or suppressor of fibrosis is dependent on the etiology of hepatic disease [9, 14, 15]. Activated HSCs also express MMPs which are endopeptidases that play an important role in degradation and removal of all of the major components of the ECM [16, 17]. The activity of MMPs is mainly regulated by tissue inhibitors of metalloproteinases (TIMPs) which are also secreted by the activated HSCs [17, 18]. Disruption in the delicate balance between MMPs and TIMPs synthesis by HSCs significantly contributes in liver fibrogenesis [17–19].

Interestingly, recent clinical studies using antiviral drugs have provided evidence about the possibility of healing/regression of fibrosis, which was long thought to be an irreversible process [2, 20]. However, effective pharmaceutical therapies and/or direct fibrolytics are still lacking and hence there is an increase demand for designing novel therapeutic strategies for established hepatic fibrosis [2, 3]. In this context, several studies have indicated the anti-fibrotic activities of a variety of natural products including vitamin D (VitD) and thymoquinone (TQ).

VitD is a steroid hormone that has traditionally been associated with systemic Ca^2+^ homeostasis and bone mineralization. Indeed, VitD regulates hundreds of different genes together with its well-established immunomodulatory and anti-inflammatory effects [21–23]. Additionally, pathological alterations in VitD concentrations have been observed with a variety of chronic liver diseases and there was a significant correlation between the levels of the hormone with the degree of liver fibrosis [24–26]. TQ is another natural product that has been shown to exhibit immunomodulatory and hepatoprotective actions and is the most abundant constituent of the *Nigella sativa* seeds, which are popularly known as black seeds or the seeds of blessing [27–29]. Despite the previous reports on the anti-fibrotic properties of VitD and TQ [21, 23, 24, 27–29], none of the previous studies explored whether dual therapy with both agents could have potential fibrolytic activities and/or boosted interactions for the treatment of liver fibrosis.

The present study therefore measured the effects of VitD and TQ dual therapy, which was initiated following the establishment of liver fibrosis by carbon tetrachloride (CCl4) in rats, and the results were compared with controls and monotherapy groups. Additionally, the effects of combining both agents on the hepatic expression of TGF-β1, IL-6, IL-10, IL-22, their receptors and MMP-9 were measured at the protein and gene levels. The exploration of possible additive interactions between VitD and TQ as anti-fibrotics and/or fibrolytics could lead to the development of more effective alternative/complementary therapeutic approaches, especially in those patients with advanced liver fibrosis and/or decompensated liver diseases.

## Methods

### Drugs and chemicals

Vitamin D3 oral drops was purchased from Novartis International AG (Basel, Switzerland), while Carbon tetrachloride, TQ, dimethylsulfoxide (DMSO) and olive oil were from Sigma-Aldrich Co. (MO, USA).

### Study design

All experimental protocols were approved by the Committee for the Care and Use of Laboratory Animals at Umm Al-Qura University and were in accordance with the EU Directive 2010/63/EU for animal experiments. A total of 85 male Wistar rats of 10 weeks of age and weighing 200–250 g each were housed in clean and sterile polyvinyl cages (5 rats/cage), maintained on standard laboratory pellet diet and water ad libitum; and kept in a temperature-controlled air-conditioned at 22–24 °C and 12 h dark/light cycle. All animals received humane care during the study protocol and during euthanasia. The animals were allocated randomly into 10 negative control rats ‘NC group’ and another 30 animals that only received CCl4 were distributed equally into short ‘S-PC’ and long ‘L-PC’ positive controls. The remaining animals were also equally divided (15 rats/group) into those that received CCl4 + VitD3 ‘VitD group’; CCl4 + TQ ‘TQ group’ and the last group received CCl4 + VitD3 + TQ ‘VitD/TQ group’.

### Treatment protocol

The total duration of the study was 16 weeks of interventions and euthanasia was carried out in the 1st day of week 17 for all groups, except the S-PC that was euthanized at the 1st day of week 9. CCl4 was prepared fresh on the day of use by mixing it with olive oil at a ratio of 1:1 and the mixture was then injected intraperitoneally in the designated groups twice weekly at a dose of 3 μl/g body weight for a total of 7 weeks. The rats in the ‘NC group’ were also injected with olive oil mixed with sterile saline during the first 7 weeks at the same time of injecting CCl4 in the remaining groups. All animals were left under observation following the last CCl4 injection and with no further intervention for one week.

Treatment with VitD and/or TQ started from week 9 on the day at which CCl4 injection would have been given and the treatment continued till the day before euthanasia. Cholecalciferol (4500 IU/ml) was prepared by adding 7.8 to 27.2 ml sterile saline every morning to form a final concentration of 1000 IU/ml and 0.5 ml (500 IU) were given orally every other day (3 days/week) to each rat in the ‘VitD’ and ‘VitD/TQ’ groups as previously described [30]. TQ (240 mg) was also freshly dissolved on the day of use in 8 ml of 0.5 % DMSO, diluted in 8 ml olive oil to prepare a final concentration of 15 mg/ml and then administered orally every other day (3 days/week) at the dose of 35 mg/kg/day (0.5 ml/rat) using gastric gavage [31]. The rats in the ‘NC’, ‘L-PC’ and ‘VitD’ groups also received a mixture of olive oil and 0.5 % of DMSO orally similar to the ‘TQ’ and ‘VitD/TQ’ groups.

### Types of samples

All rats were euthanized following anaesthesia using diethyl ether (Fisher Scientific UK Ltd, Loughborough, UK) and 3 ml of venous blood were collected from each animal in a plain tube. All blood samples were centrifuged and the serum was used to measure the levels of liver enzymes (ALP, ALT and AST), renal function parameters (creatinine, BUN and urea) and concentrations of 25-OH vitamin D on Cobas e411 (Roche Diagnostics International Ltd, Switzerland) according to the manufacturer’s protocol.

For histopathology and immunohistochemistry experiments, a liver specimen of 1 cm length ×0.5 cm width ×0.5 thickness was taken from the middle lobe of each liver, processed by a conventional method and finally embedded in paraffin. Another two specimens from the middle lobe of the liver (1 gm/each) were also obtained from each animal with one piece being immediately processed for protein extraction using 6 ml of RIPA lysis buffer containing protease inhibitors (Santa-Cruz Biotechnology Inc., CA, USA) and electrical homogeniser. Following centrifugation, the concentrations of total proteins were measured at 280 OD on a BioSpec-nano machine (Shimadzu Corporation, Tokyo, Japan). All samples were then diluted by normal sterile saline for a final concentration of 500 µg/ml of total protein to measure the levels of candidate proteins in liver by ELISA.

The second specimen was immediately immersed in 15 ml of RNA*Later* (Thermo Fisher Scientific, CA, USA) and total RNA was later extracted using the Purelink RNA mini kit (Thermo Fisher Scientific) according to the manufacturer’s instructions and following homogenization using tissue raptor and sterile plastic tubes with beads (Omni International, GA, USA). The quality and quantity of extracted RNA was assessed on a BioSpec-nano machine and typically had an A260/A280 ratio of 1.7–1.9 and cDNA was immediately synthesized by transcribing 200 ng of total RNA using a high capacity RNA-to-cDNA Reverse Transcription Kit (Thermo Fisher Scientific) according to the manufacturer’s protocol.

### Histology studies

Serial sections of 5 μm thickness were cut from each tissue block and were stained with haematoxylin and eosin (H&E) and Mason’s trichrome (Abcam, MA, USA) to assess hepatic architecture and collagen type I deposition, respectively. All sections were examined on an EVOS XL Core microscopy (Thermo Fisher Scientific) at ×100, ×200 and ×400 magnifications by two expert histopathologists who were blind to the source groups to evaluate and score liver fibrosis in 20 random fields at ×200 and according to the previously published scaling system as follow: 0 = Absent; 1 = Slight; 2 = Moderate; 3 = Severe and 4 = cirrhosis [32, 33]. Additionally, quantitative measurement of collagen deposition (fibrosis index  %) was done using ImageJ software (https://imagej.nih.gov/ij/) as previously described [33, 34] (Additional file 1: Figure S1).

### Enzyme linked immunosorbant assay (ELISA)

The concentrations of TGF-β1, IL-6, IL-10, IL-22 and MMP-9 proteins in liver tissue homogenates were measured using specific rat ELISA kits (R&D systems, Minneapolis, USA). All samples were processed in duplicate on a fully automated ELISA system (Human Diagnostics, Germany) and according the manufacturers’ guidelines. As reported by the manufacturer, the detection range of both TGF-β1 and IL-10 kits was between 31.2 and 2000 pg/ml, with sensitivities of 4.6 pg/ml for TGF-β1 and 10 pg/ml for IL-10, intra-assay and inter-assay precisions of <4 and <10 % for both kits. The IL-6 kit had a detection range between 62.5 and 4000 pg/ml with a sensitivity of 21 pg/ml, intra-assay precision <8.8 % and inter-assay precision <10 %. The detection range of the IL-22 kit was 15.6–1000 pg/ml, a sensitivity of 8.2 pg/ml, intra-assay precision <5 % and inter-assay precision <10 %. The kit for MMP-9 detects the total protein (free and bound) with a range between 0.2 and 10 ng/ml, sensitivity of 0.028 ng/ml and, both intra-assay and inter-assay precisions <6.9 %.

### Immunohistochemistry

The primary antibodies (Santa-Cruz Biotechnology Inc.) against IL-6, IL-10 A & B and IL-22A1 receptors were polyclonal goat IgG antibodies while rabbit polyclonal IgG antibodies were used to detect TGF-β type II and IL-22A2 receptors. An avidin–biotin horseradish peroxidase technique was applied to localize the target molecules by ImmunoCruz™ Rabbit or Goat LSAB Staining Systems (Santa-Cruz Biotechnology Inc.) according to the manufacturer’s protocol. The concentrations were 1:200 for TGF-βRII and IL-6RA antibodies, and 1:100 for the remaining antibodies. The negative control slides consisted of a section of the tissue block being studied that was treated identically to all other slides but the primary antibodies were replaced with corresponding primary normal goat or rabbit IgG antibodies (Santa-Cruz Biotechnology Inc.) to control for non-specific staining.

The sections were observed on an EVOS XL Core microscope to evaluate and score the immunostain. Each section was examined by two observers who were blind to the source of sections and the intensity of staining was assessed in 5 random fields of each section at ×200 magnification and by using ‘H score’ that was calculated as follow [30, 35]: H score = ƩP_ί_ (ί +1), where ί represents the intensity of staining (0 = negative; 1 = weak; 2 = moderate and 3 = strong) and P_ί_ is the percentage of cells (0–100 %) stained at each intensity. In the case of a wide disagreement between both observers, the slides were reanalyzed by a third independent reviewer.

### Quantitative RT-PCR

The PCR reactions were carried out in triplicate wells on ABI® 7500 system using power SYBR Green master mix (Thermo Fisher Scientific). Each PCR well included 10 µl SYBR Green, 7 µl DNase/RNase free water, 1 µl containing 5 pmol of each primer (Additional file 2: Table S1) and cDNA (25 ng) and, 40 cycles (95 °C/15 s and 60 °C/1 min) of amplification were performed. Negative controls included one minus-reverse transcription (minus-RT) control from the previous RT step and another minus-template PCR, in which nuclease free water was used as a template.

The 2^−∆∆Ct^ method was used to perform relative quantitative gene expression of rat *TGFB1*, *TGFBR2*, *IL6*, *IL6RA*, *IL10*, *IL10RA*, *IL10RB, IL22*, *IL22RA1*, *IL22RA2* and *MMP9* target genes. Three reference genes were tested and rat *β*-*actin* gene showed the most consistent results and it was used to normalize the Ct values of the genes of interest. The results are expressed as fold-change compared with the ‘NC group’.

### Statistical analysis

Statistical analysis of the results was performed using SPSS version 16. Normality and homogeneity of data were assessed with the Kolmogorov and Smirnoff test and Levene test, respectively. Student’s t test or Mann–Whitney U test was used to compare between 2 groups based on data normality. One way ANOVA followed by LSD post hoc test or Kruskal–Wallis followed by Dunn’s post hoc test were used to compare between >2 groups depending on the data homogeneity. P value <0.05 was considered significant.

## Results

### Biochemical findings

There was no significant difference (P > 0.05) between the study groups in renal function parameters (Table 1). However, serum ALP (P = 0.003), ALT (P = 0.04 × 10^−5^) and AST (P = 0.0005) enzymes were significantly increased in in the ‘S-PC’ than the ‘NC’ group (Table 1). A further significant increase in the 3 enzymes (P = 0.07 × 10^−4^; P = 0.0001 and P = 0.004, respectively) was also detected in the ‘L-PC’ compared with ‘S-PC’. Remedial monotherapy with TQ or VitD significantly decreased (P < 0.05) the serum levels of the liver enzymes compared with the 2 positive control groups. Nevertheless, the levels remained significantly higher, except for AST, following single therapy with TQ (P = 0.008 for ALP; P = 0.02 for ALT) and VitD (P = 0.02 for ALP; P = 0.03 for ALT) compared with ‘NC’ group (Table 1). Dual therapy with VitD and TQ resulted in the most significant decrease in the liver enzymes compared with both positive control groups and the results were comparable to those of NC group (Table 1). Additionally, the serum 25-OH vitamin D concentrations were significantly higher (P < 0.05) in the groups that received cholecalciferol compared with the other study groups. However, there was no significant difference between the study groups in the serum calcium concentrations (Table 1).

**Table 1:** Mean ± SD of body weight, serum concentrations of 25-OH vitamin D, calcium, liver enzymes and renal function parameters in the different study groups

	NC group	S-PC group	L-PC group	TQ group	VitD group	VitD/TQ group
Body weight (g)	231.57 ± 20.01	222.8 ± 18.71	201.97 ± 23.01^a,b^	228.42 ± 13.64^c^	230.1 ± 22.2^c^	229.5 ± 18.7^c^
25-OH Vitamin D (ng/ml)	46.19 ± 8.1	34.3 ± 4.9^a^	26.6 ± 6.7^a,b^	35.7 ± 9.5^a,c^	65.5 ± 9.1^a,b,c,d^	68.8 ± 8.7^a,b,c,d^
Calcium (mg/dL)	9.17 ± 0.51	9.1 ± 0.63	9.22 ± 0.44	9.24 ± 0.35	9.29 ± 0.42	9.23 ± 0.38
ALP (IU/L)	122.6 ± 11.2	211.4 ± 23.8^a^	315.7 ± 29.7^a,b^	170.8 ± 19.4^a,b,c^	167.3 ± 21.9^a,b,c^	131.6 ± 21.6^b,c^
ALT (U/L)	67 ± 5.4	133.3 ± 12.4^a^	271.2 ± 18.7^a,b^	88.7 ± 13.1^b,c^	75.4 ± 7.3^b,c^	69.3 ± 8.7^b,c,d^
AST (U/L)	92.4 ± 24.2	166.1 ± 28.8^a^	218.8 ± 26.7^a^	109.2 ± 21.6^b,c^	103 ± 19.8^b,c^	99 ± 11.9^b,c^
Creatinine (mg/dL)	0.22 ± 0.03	0.23 ± 0.05	0.2 ± 0.06	0.2 ± 0.03	0.19 ± 0.03	0.21 ± 0.05
Urea (mg/dL)	47.6 ± 5.1	49.1 ± 4.8	52.3 ± 4	51.6 ± 9.5	47.3 ± 5.8	49.1 ± 3.7
BUN (mg/dL)	22.2 ± 2.4	22.7 ± 2.8	24.4 ± 1.9	23.3 ± 2.4	22 ± 2.7	21.9 ± 2.4

^a^P < 0.05 compared with normal ‘NC group

^b^P < 0.05 compared with short positive control ‘S-PC group’

^c^P < 0.05 compared with long positive control ‘L-PC group’

^d^P < 0.05 compared with ‘TQ group’

^e^P < 0.05 compared with ‘VitD group’

### Effects of vitamin D3 and TQ mono and dual therapies on liver fibrosis

**Fig. 1:**
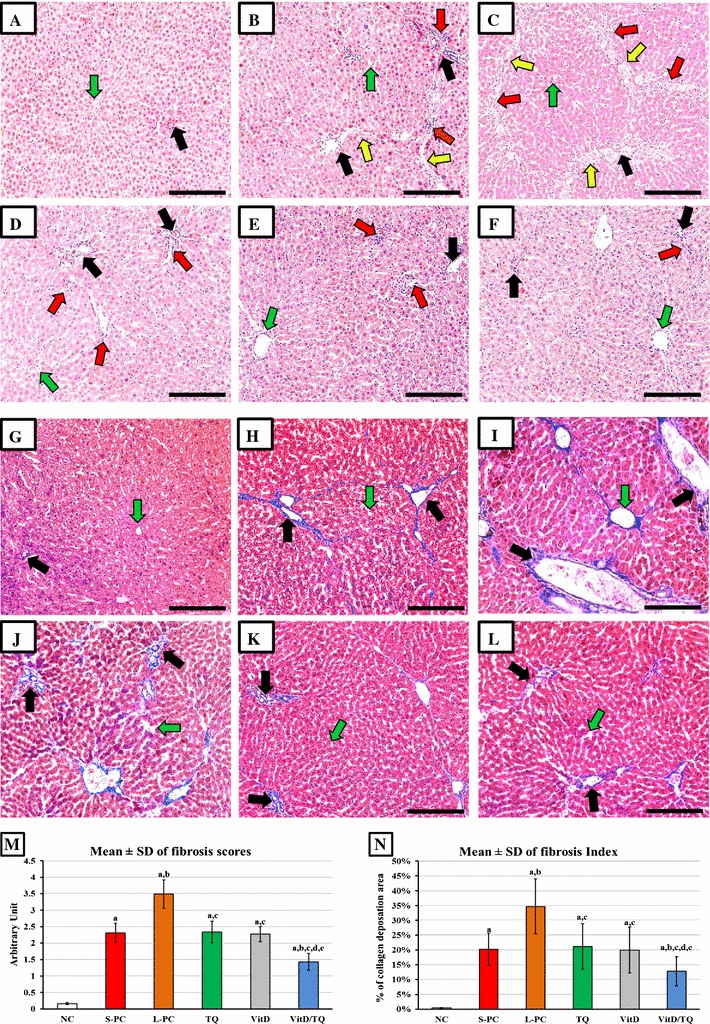
Histopathological features of hepatic sections from the NC (**A**, **G**), S-PC (**B, H**), L-PC (**C**, **I**), TQ monotherapy (**D**, **J**), VitD monotherapy (**E**, **K**) and VitD/TQ dual therapy (**F**, **L**) groups by H&E (**A–F**) and Masson’s trichrome (**G–L**) stains (×200 magnification, *scale bar* = 8 µm). Additionally, the (**M**) arbitrary fibrosis score and (**N**) fibrosis index are shown in the bar graphs between the study groups. (*Green arrow* = central vein; *black arrow* = portal tract; *red arrow* = cellular infiltrations; *yellow arrow* = fibrous septa; a = P < 0.05 compared with NC group; b = P < 0.05 compared with S-PC group; c = P < 0.05 compared with L-PC group; d = P < 0.05 compared with TQ group and e = P < 0.05 compared with VitD group)

H&E stained liver specimens from the NC-group showed normal features of hepatocytes, liver architecture, central veins and portal areas (Fig. 1A). CCl4 injection resulted in distortions of the normal hepatic architecture with swollen hepatocytes around the central veins and interruption of hepatic tissue by numerous fibrous septa together with diffuse cellular infiltration around the fibrous bands and dilated portal areas in the S-PC (Fig. 1B) and L-PC (Fig. 1C) groups. Furthermore, more prominent damage with excessive hepatic lobulation was evident in the L-PC group. Monotherapy with TQ (Fig. 1D) or VitD (Fig. 1E) was associated with better histological features and lower cellular infiltration in addition to less fibrous septa compared with L-PC. However, no major differences were observed between both monotherapy groups or by comparing each of them with the S-PC. Interestingly, dual therapy with TQ and VitD (Fig. 1F) showed the best improvement in the hepatic microanatomy compared with all other CCl4 groups with more marked restoration towards normal hepatic histology and a reduction of cellular infiltration but the hepatocytes were still swollen around the dilated central veins.

Minimal/negligible deposition of collagen type I was observed around the portal areas in the NC-group by Masson’s trichrome stain (Fig. 1G). Additionally, portal fibrosis and peri-portal septa were evident in both the S-PC (Fig. 1H) and L-PC (Fig. 1I) specimens and both groups had significantly higher fibrosis score (Fig. 1M; P = 0.02 × 10^−5^ and P = 0.04 × 10^−8^, respectively) and index (Fig. 1N; P = 0.05 × 10^−7^ and P = 0.03 × 10^−11^, respectively). However, the fibrogenesis process was significantly more progressive in the L-PC specimens reaching to cirrhosis, which was characterized by large fibrous bands extending from the portal areas to the central veins through the hepatic tissue, resulting in extensive abnormal lobulation of the examined sections. TQ (Fig. 1J) and VitD (Fig. 1K) monotherapies significantly inhibited the progression of fibrogenesis compared with the L-PC group but fibrosis was yet observed around the portal areas with peri-portal fibrous extensions similar to the S-PC group. Additionally, the fibrosis score (P = 0.6) and index (P = 0.8) were similar between both groups of single therapy and none of them showed significant difference (P > 0.05) compared with S-PC group. In parallel with the H&E observations, combined therapy of TQ and VitD (Fig. 1L) showed the best mitigation in liver damage that was indicated by a significant regression in the amount of fibrous tissues that was mainly restricted around the portal areas. The fibrosis score and index were also significantly lower than S-PC (P = 0.003 and P = 0.02, respectively), L-PC (P = 0.04 × 10^−4^ and P = 0.03 × 10^−6^, respectively), TQ (P = 0.0009 and P = 0.03, respectively) and VitD (P = 0.002 and P = 0.02, respectively) groups. Nevertheless, the fibrosis score and index were still significantly higher than normal hepatic specimens (Fig. 1M, N).

### Hepatic tissue concentrations of targeted proteins

The concentrations of TGF-β1 (P = 0.004), IL-6 (P = 0.04 × 10^−3^) and IL-22 (P = 0.008) increased significantly in parallel with significant decreases in IL-10 (P = 0.01) and MMP-9 (P = 0.03), proteins in the tissue homogenate samples from S-PC compared with NC (Table 2). Additional significant alterations in the hepatic concentrations of TGF-β1 (P = 0.02), IL-10 (P = 0.03), IL-22 (P = 0.005), and MMP-9 (P = 0.007), but not IL-6 (P = 0.4), were also detected between L-PC and S-PC groups (Table 2).

**Table 2:** Mean ± SD of protein concentrations of TGF-β1, IL-6, IL-10, IL-22 and total MMP9 in liver tissue homogenates by ELISA

	NC group	S-PC group	L-PC group	TQ group	VitD group	VitD/TQ group
TGF-β1 (pg/ml)	569.6 ± 97.8	988.4 ± 93.3^a^	1394.7 ± 101.4^a,b^	875.2 ± 76.7^a,b,c^	705.6 ± 56.3^a,b,c,d^	600.2 ± 78.7^b,c,d,e^
IL-6 (pg/ml)	663.9 ± 76.5	1721.2 ± 325.3^a^	1980.4 ± 233.4^a^	717.4 ± 110^b,c^	672.3 ± 94.9^b,c^	457.4 ± 121^a,b,c,d,e^
IL-10 (pg/ml)	479.06 ± 21.9	222.7 ± 53.8^a^	121.5 ± 26.6^a,b^	206.9 ± 23.3^a,c^	237.1 ± 25.7^a,c^	323.2 ± 33.5^a,b,c,d,e^
IL-22 (pg/ml)	379.7 ± 48.4	744.9 ± 61.7^a^	963.8 ± 102^a,b^	649.8 ± 52.3^a,b,c^	663.6 ± 47.4^a,c^	452.5 ± 38.9^a,b,c,d,e^
MMP-9 (ng/ml)	4.1 ± 0.13	3.4 ± 0.31^a^	2.6 ± 0.52^a,b^	3.7 ± 0.36^a,c^	3.9 ± 0.17^a,b,c^	4.52 ± 0.34^a,b,c,d,e^

^a^P < 0.05 compared with NC group

^b^P < 0.05 compared with S-PC group

^c^P < 0.05 compared with L-PC group

^d^P < 0.05 compared with TQ group

^e^P < 0.05 compared with VitD group

In comparison with the S-PC and L-PC groups, single therapy with TQ significantly decreased the concentrations of TGF-β1 (P = 0.002 and P = 0.01 × 10^−3^, respectively), IL-6 (P = 0.03 × 10^−5^ and P = 0.07 × 10^−7^, respectively) and IL-22 (P = 0.003 and P = 0.05 × 10^−3^, respectively). However, TQ monotherapy showed non-significant reduction in the concentrations of IL-10 (P = 0.3) and MMP-9 (P = 0.1) compared with S-PC group. In contrast, significant differences were observed between TQ and L-PC (P = 0.004 for IL-10 and P = 0.01 for MMP-9) groups.

Similarly, VitD monotherapy resulted in a decrease in TGF- β1 (P = 0.04 × 10^−3^ and P = 0.03 × 10^−5^), IL-6 (P = 0.01 × 10^−7^ P = 0.03 × 10^−11^) and IL-22 (P = 0.09 and P = 0.003) compared with the S-PC and L-PC groups, respectively (Table 2). Moreover, an increase in MMP-9 (P = 0.003 and P = 0.06 × 10^−4^) and IL-10 (P = 0.2 and P = 0.0004) was seen in the VitD group compared with both positive control groups, respectively. The lowest decrease in the liver concentrations of TGF-β1 (P = 0.09 × 10^−4^ and P = 0.006), IL-6 (P = 0.003 and P = 0.002) and IL-22 (P = 0.005 and P = 0.003) proteins, while the utmost increase in IL-10 (P = 0.002 and P = 0.004) and MMP-9 (P = 0.04 × 10^−4^ and P = 0.0001), were detected in the dual therapy group compared with TQ and VitD monotherapy groups, respectively (Table 2).

### Immunohistochemistry of targeted cytokine receptors

**Fig. 2:**
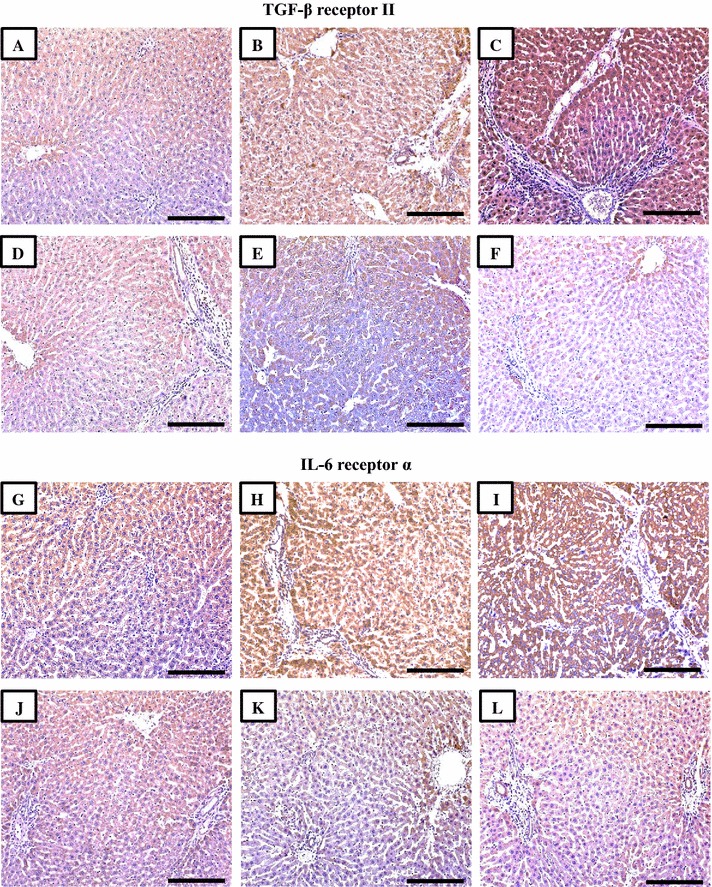
Immunohistochemical expression of TGF-β receptor II and IL-6 receptor in liver sections from the NC (**A**, **G**), S-PC (**B, H**), L-PC (**C**, **I**), TQ monotherapy (**D**, **J**), VitD monotherapy (**E**, **K**) and VitD/TQ dual therapy (**F**, **L**) groups (×200 magnification, *scale bar* = 8 µm)

**Fig. 3:**
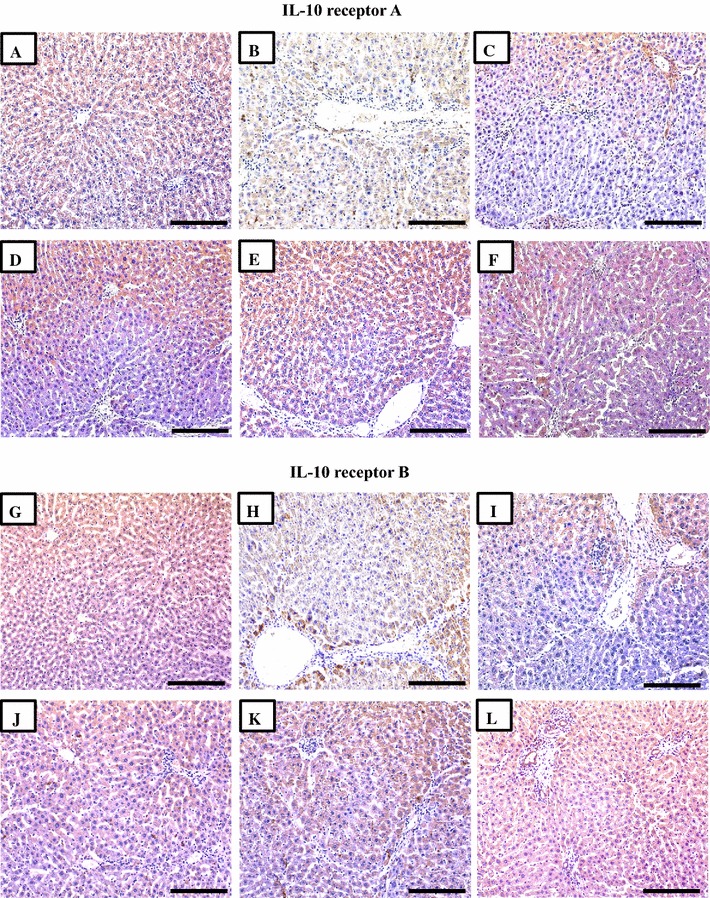
Immunohistochemical expression of IL-10 receptors A and B in liver sections from the NC (**A**, **G**), S-PC (**B, H**), L-PC (**C**, **I**), TQ monotherapy (**D**, **J**), VitD monotherapy (**E**, **K**) and VitD/TQ dual therapy (**F**, **L**) groups (×200 magnification, *scale bar* = 8 µm)

**Fig. 4:**
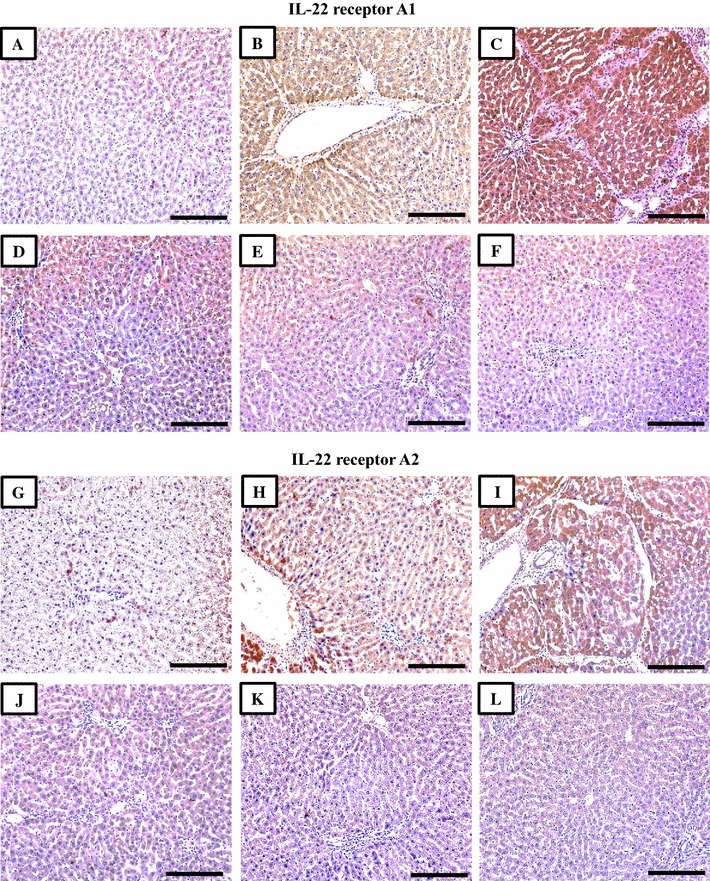
Immunohistochemical expression of IL-22 receptors A1 and A2 in liver sections from the NC (**A**, **G**), S-PC (**B, H**), L-PC (**C**, **I**), TQ monotherapy (**D**, **J**), VitD monotherapy (**E**, **K**) and VitD/TQ dual therapy (**F**, **L**) groups (×200 magnification, *scale bar* = 8 µm)

All target receptors were localized mainly at the cell membrane and occasionally in the cytoplasm of normal liver cells from the NC group (Figs. 2A, G, 3A, G, 4A, G). Following establishment of fibrosis by CCl4, the intensity of the immunostain was significantly stronger for TGF-βRII (Fig. 2B; P = 0.006), IL-6R (Fig. 2H; P = 0.0003), IL-22RA1 (Fig. 4B; P = 0.04 × 10^−4^) and IL-22RA2 (Fig. 4H; P = 0.01 × 10^−5^) in the S-PC compared with NC group (Table 3). In contrast, a significant decrease in the expression of IL-10 receptors A (P = 0.007) and B (P = 0.03) was observed between the S-PC (Fig. 3B, H respectively) and NC groups (Table 3). Furthermore, a significantly stronger staining was noted in TGFβRII (Fig. 2C; P = 0.008), IL6R (Fig. 2I; P = 0.03) and IL22R A1 (Fig. 4C; P = 0.09 × 10^−4^) and A2 (Fig. 4I; P = 0.03) between the L-PC and the S-PC groups. Concurrently, weaker significant expressions were detected, mainly adjacent to fibrotic areas, in IL10 receptors A (Fig. 3C; P = 0.03) and B (Fig. 3I; P = 0.009) between both groups.

**Table 3:** Mean ± SD of immunohistochemistry scores for TGF-β receptor II, IL-6 receptor, IL-10 receptors type A and type B, and IL-22 receptors type A1 and A2 in liver specimens from the different study groups

	NC group	S-PC group	L-PC group	TQ group	VitD group	VitD/TQ group
TGF-β receptor II	184.4 ± 28.7	297.9 ± 22.3^a^	345.2 ± 24.2^a,b^	266.7 ± 23.6^a,b,c^	242.2 ± 26.8^a,b,c^	177.2 ± 17.8^b,c,d,e^
IL-6RA	201 ± 26.7	319.6 ± 22.3^a^	370.6 ± 28.7^a,b^	287.6 ± 25.3^a,b,c^	229.6 ± 30.1^a,b,c,d^	169.7 ± 22.6^b,c,d,e^
IL-10RA	163.5 ± 31.2	86.4 ± 23.4^a^	58.6 ± 19.3^a,b^	158.6 ± 24.3^b,c^	171.9 ± 28.5^b,c^	228.1 ± 27.8^a,b,c,d,e^
IL-10RB	219.1 ± 23.8	147.1 ± 28.8^a^	103.2 ± 20.3^a,b^	226.1 ± 23.9^b,c^	215.7 ± 31.6^b,c^	231.3 ± 22.2^b,c^
IL-22RA1	122.4 ± 29.5	256.9 ± 32.3^a^	373.1 ± 26.8^a,b^	211.8 ± 37.2^a,b,c^	178.1 ± 33.6^a,b,c,d^	153.1 ± 21.6^a,b,c,d^
IL-22RA2	87.7 ± 18.6	311.4 ± 29.9^a^	366.5 ± 22.6^a,b^	237.4 ± 26.8^a,b,c^	194.7 ± 28.6^a,b,c,d^	168 ± 25.4^a,b,c,d,e^

^a^P < 0.05 compared with NC group

^b^P < 0.05 compared with S-PC group

^c^P < 0.05 compared with L-PC group

^d^P < 0.05 compared with TQ group

^e^P < 0.05 compared with VitD group

In agreement with the results of ELISA, single therapy with TQ or VitD lead to a significant decrease in the expression of TGF-βRII (Fig. 2D; P = 0.03 and Fig. 2E; P = 0.002, respectively), IL-6R (Fig. 2J; P = 0.03 and Fig. 2K; P = 0.002, respectively), IL-22RA1 (Fig. 3D; P = 0.02 and Fig. 3E; P = 0.008, respectively), IL-22RA2 (Fig. 3J; P = 0.03 and Fig. 3K; P = 0.002, respectively) compared with the S-PC group (Table 3). Additionally, there was a significant increase in the intensity of IL-10RA (Fig. 2D; P = 0.02 and Fig. 2E; P = 0.006, respectively) and IL-10RB (Fig. 3K; P = 0.02 and Fig. 3L; P = 0.03, respectively) in the TQ and VitD groups compared with the S-PC group (Table 3). Significant differences (P < 0.05) were also observed between the two monotherapy groups in the intensity of immunostain for all receptors except IL10RA (P = 0.2) and IL10RB (P = 0.4).

By further analysis, the dual therapy group showed the most significant alterations in the expression of TGFBRII (P = 0.03 × 10^−4^ and P = 0.05 × 10^−5^), IL-6R (P = 0.0002 and P = 0.07 × 10^−6^), IL-10RA (P = 0.03 and P = 0.007), IL-22RA1 (P = 0.004 and P = 0.1) and IL-22RA2 (P = 0.004 and P = 0.0002), compared with TQ monotherapy and VD monotherapy groups, respectively. However, no difference was observed in IL10RB between the 3 groups (Table 3).

### Hepatic gene expression of targeted molecules and their receptors

**Fig. 5:**
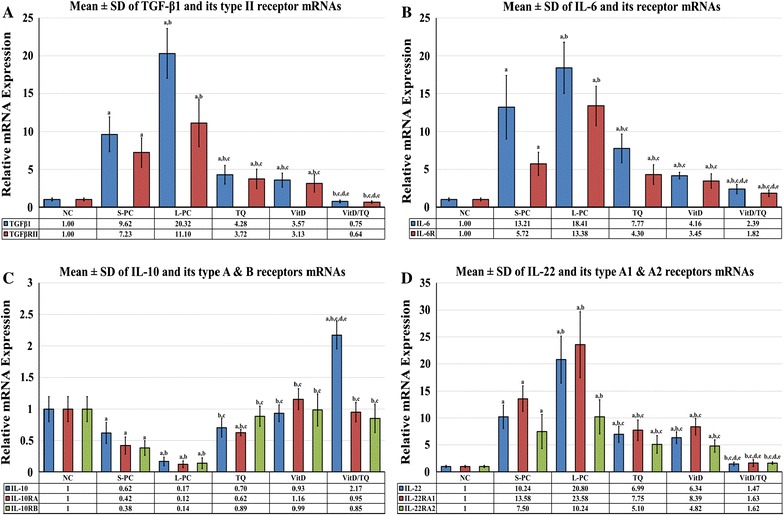
Mean ± SD of messenger RNA relative expression of **A** TGF-β1 and its type II receptor, **B** IL-6 and its receptor, **C** IL-10 and its type **A** and **B** receptors and **D** IL-22 and its type A1 and A2 receptors in the different study groups. (a = P < 0.05 compared with NC group; b = P < 0.05 compared with S-PC group; c = P < 0.05 compared with L-PC group; d = P < 0.05 compared with TQ group and e = P < 0.05 compared with VitD group)

The results of quantitative RT-PCR experiments showed a significant decrease in the mRNA expression of *MMP9* in the S-PC (five folds; P = 0.01 × 10^−6^) and L-PC (nine folds; P = 0.03 × 10^−9^) compared with the NC group. Single therapy with TQ or VitD induced a significant up-regulation in *MMP9* mRNA compared with S-PC (~4 folds; P = 0.0004 and 3.2 folds; P = 0.002, respectively) and L-PC groups (sevenfolds; P = 0.03 × 10^−4^ and 5.8 folds; P = 0.002, respectively). The highest increase in the *MMP9* mRNA was detected in the dual therapy compared with TQ (1.6 folds; P = 0.006) and VitD (2.1 folds; P = 0.004) monotherapy. The results of the remaining target molecules correlated with their corresponding proteins results and are illustrated in Fig. 5.

## Discussion

Herein, the anti-fibrotic and fibrolytic activities of remedial therapies with TQ and VitD individually or combined were measured in established liver fibrosis by CCl4 in rats. We also measured whether combining both agents could have additive/enhanced mechanistic effects on a panel of pro- and anti-fibrogenesis mediators. The histopathological findings confirmed the development of fibrosis prior to the initiation of the different remedial therapy protocols and liver damage was significantly more aggravated and reached to cirrhosis in the L-PC group compared with S-PC. TQ or VitD as single therapy lead to a significant restoration of hepatic architecture as well as a significant decrease in the deposition of ECM compared with the L-PC but not the S-PC group. Additionally, there were no significant differences in the fibrosis score and index between the two monotherapy protocols. At the molecular level, single therapy with TQ or VitD significantly decreased the genes and proteins expression of TGF-β1, IL-6, IL-22 and their corresponding receptors, while increased the levels of MMP-9, IL-10 and its receptors compared with both S-PC and L-PC groups.

These observations are in agreement with several previously published results from a variety of studies that have shown that TQ and VitD mitigate the development of fibrosis through their immunomodulatory actions [22, 26–28]. However, the absence of a significant alleviation in liver fibrosis between the TQ, VitD and the S-PC groups suggest that monotherapy with any of these agents of interest could be an effective remedial strategy for the prevention of further progression, but not fibrolysis, of a previously established liver fibrosis.

Nevertheless and to the best of our knowledge, this study is the first to measure the effect of TQ and VitD combined therapy for the treatment of liver fibrosis. The present data showed that dual therapy resulted in a significant resolution of fibrosis that concurred with a reduction in the serum levels of liver enzymes compared with S-PC group. Furthermore, the dual therapy resulted in the most significant downregulations as well as upregulations of the tested pro- and anti-fibrogenic pathways, respectively. Thus we speculate that, in addition to their well-established anti-fibrotic effects, the combination of the two nutraceuticals could represent a novel and potentially effective direct fibrolytic strategy against established liver fibrosis.

Hepatic fibrogenesis involves multiple cellular and molecular events. Following liver injury, stimulated Kupffer cells secret TGF-β1, which in turn activates HSCs, and later the deposition of excess ECM [2, 3, 22]. Activated HSCs also produce higher amounts of IL-6 that increases collagen production, HSCs proliferation and differentiation, and neutralizes the activities of MMPs [17–20] by upregulating the production of TIMPs [13, 36]. Contrarily, IL-10 is believed to inhibit the fibrogenic process by increasing the production of immunoglobulins, downregulating pro-fibrotic cytokines and promoting the degradation of ECM [7, 37, 38].

Our results correlate with the previous reports since they showed a significant increase in TGF-β1, IL-6 and their receptors with a simultaneous significant decrease in MMP-9, IL-10 and its receptors at the protein and gene levels in the positive control groups. Additionally, the development of liver fibrosis in our study was associated with significantly higher levels of IL-22 ant its receptors, suggesting a pro-fibrogenic role for the cytokine [9–12]. The effect of hepatic IL-22 as whether pro- or anti-fibrotic is poorly understood, the available data are conflicting and the role of the cytokine in liver fibrogenesis appears to be dependent on the etiological context of liver disease [9, 14, 15]. In this regards, several earlier studies have reported an increase in Th-17 and IL-22 during fibrogenesis [9–13] and in vitro treatment with IL-22 also stimulated HSCs to secrete pro-fibrotic cytokines [12]. On the other hand, others also demonstrated that IL-22 had a hepatoprotective role and alleviated the development of hepatic fibrosis [15, 39, 40]. Therefore, further in vivo studies using several models of liver fibrosis are required to illustrate the precise role of IL-22 in the pathogenesis of liver fibrosis.

VitD is acquired as a pro-hormone mainly following skin exposure to sun light and, to a lesser extent, from nutritional sources [41]. The pro-hormone is later converted in the liver by the actions of the 25-hydroxylase enzyme to an intermediate form [25(OH)VitD], which is subsequently transported to the kidney for a final activation step by the 1-α hydroxylase enzyme [41]. Chronic liver or kidney diseases are therefore well-known causes of abnormal low levels of the hormone and the findings of several studies have demonstrated beneficial effects of adding VitD supplementation during the course of treatment of hepatic and renal diseases [21, 42]. The present study is in support to the previous observations since oral supplementation with cholecalciferol resulted in a restoration in the observed decrease in serum levels of 25-OH VitD following hepatic damage by CCl4. Notably, TQ monotherapy, possibly through its hepatoprotective properties [43, 44], also resulted in a significant alleviation in serum 25-OH VitD levels compared with L-PC group. However, more studies are needed to measure the effects of TQ on the activities of hepatic 25-hydroxylase enzyme to support our observations.

The hepatoprotective and anti-fibrogenic effects of both TQ and VitD have been previously shown and VitD is the most studied among both compounds in liver fibrosis. Animal studies have demonstrated that chronic vitamin D deficiency or the deletion of *Vdr*^−*/*−^ gene in mice lead to spontaneous development of liver fibrosis with abnormal increase in the levels of TGF-β1 and IL-6 as well as a significant decrease in IL-10 [45, 46]. The expression of VDR also decreased significantly in activated primary rat HSCs in vitro, an effect that was reversed by exogenous treatment with VitD [22]. The researchers have further reported that the production of collagen Iα1 was VDR dependent and, treatment with VitD also lead to up-regulation of MMP-9, cell cycle arrest and inhibition of HSCs proliferation [22]. Similar results have also been shown by more recent studies on cultured primary HSCs from mice [47] and human [48]. Additionally, a significant negative correlation has also been observed between the serum levels of vitamin D either with serum IL-6 or with the degree of liver damage in patients with chronic hepatitis C [21, 25, 26].

Equivalent results have also been reported following the use of TQ for the treatment of liver fibrosis in a variety of experimental in vivo models as well as in vitro experiments [29, 43, 49]. TQ therapy was also associated with inhibition of the trans-differentiation process of immortalized human HSCs and reduction of ECM deposition together with a significant increase in the levels of IL-10 and MMPs [43, 44]. Our findings are aligned and support the aforementioned studies as there was a significant resolution in hepatic fibrosis and liver enzymes following monotherapy with either VitD or TQ compared with L-PC group. Additionally, both compounds resulted in a significant decrease in the tissue concentrations of TGF-β1, IL-6, IL-22 as well as the expression of their corresponding receptors at the gene and protein levels. A significant increase in the hepatic concentrations of MMP-9 and IL-10 system was also observed in the groups treated with VitD and TQ. Nevertheless, there was no significant difference between both agents, as well as when compared with the S-PC group, in the degree of liver damage induced by CCl4. These findings provide additional support to the notions that monotherapy with either TQ [43, 44] or VitD [45, 46] could be an efficient nutraceutical strategy for the prevention and/or protection from further liver damage induced by chronic hepatic inflammation. However, our results also suggest that the monotherapy with either of the agents of interest has a limited efficacy in the resolution of an established liver fibrosis.

At the present time, little is known regarding whether VitD or TQ could have direct fibrolytic activities. In this concern, all the available studies that investigated the effects of TQ initiated the therapeutic protocol either before or at the same time of fibrogenesis induction and none of them tested the compound following the establishment of liver fibrosis [29, 43, 49]. On the other hand, only a single study measured the anti-fibrogenic as well as fibrolytic effects of VitD following thioacetamide and bile duct ligation models, respectively [23]. Similar to our observations, the researchers have reported that VitD inhibited the development of fibrosis but did not induce significant regression in established cirrhosis [23]. Notably, ours is the first study to demonstrate a significant regression in liver fibrosis and ECM deposition following TQ and VitD dual therapy, compared with all other CCl4 injected groups, including S-PC. We therefore hypothesis that the dual therapeutic strategy with TQ and VitD could have promising direct fibrolytic activities in the liver.

The breakdown of ECM is mainly achieved through MMPs, which are tightly regulated by TIMPs. The latter bind reversibly to the active site of MMPs in a 1:1 molar ratio and, pathological imbalances in this system have been linked to the pathogenesis of hepatic fibrosis [17, 18]. It has also been shown that the levels of TIMPs increase significantly by TGF-β, IL-6 and IL-22 in several in vitro and in vivo studies [16, 50, 51]. Alternately, treatment with IL-10 has been demonstrated to significantly increase the production of MMPs, including MMP-9, as well as significantly decrease TIMPs during liver fibrosis [7, 37, 38].

Both TQ and VitD have been reported to upregulate the protein expression and activity of many MMPs; among them is MMP-9, and to suppress the expression of TIMP-1 [23]. MMP-9 is known for its high affinity to bind with TIMP-1 thus scavenging and reducing the effects of TIMP-1 on the other MMPs [18, 52]. Additionally, the observed fibrolytic effect of combined therapy protocol in our study was associated with improved actions for TQ and VitD on the expression of TGF-β1, IL-6, IL-10, IL-22 and MMP-9 in hepatic tissue. Hence, we propose that dual therapy with VitD and TQ could directly stimulate the degradation of ECM by inducing the production of MMPs and/or downregulating TIMPs through the modulation of several cytokines. However, future studies to measure the effect of TQ and VitD combined therapy on the expression of MMPs and TIMPs in different models of liver fibrosis as well as by HSCs in vitro are essential to support our hypothesis.

## Conclusions

VitD and TQ modulated several pro- and anti-fibrogenic pathways and exhibited comparable anti-fibrotic effects in CCl4 model of liver fibrosis. Additionally, their combination resulted in enriched and significant mitigation of previously established liver fibrosis and might offer a potential direct fibrolytic strategy. Further studies are need to illustrate the clinical value of both natural products in the treatment of liver fibrosis in human.

## Additional files

10.1186/s12967-016-1040-4 Steps of processing the study digital images with ImageJ software for calculating the fibrosis index (% of collagen deposition), which was identified by blue staining, following Masson’s trichrome stain of liver sections from S-PC (1st upper row), L-PC (2nd row), TQ (3rd row), VitD (4th row) and dual therapy (bottom row) groups. The identification and selection of the areas of interest (left column) were done with the guidance of an expert histopathologist. The images were then processed by hue/saturation/brightness (HSB) for color threshold adjustment using ‘black’ as the threshold color to digitally mark and select an area of interest by the software (middle column). This was followed by clearing outside the defined areas of interest to ensure the precision of the identification and selection processes by the software (Right column). All measurements were calculated following calibration with digital photos of corresponding microscopic scale slides captured at the designated magnifications. (×200 magnification, scale bar = 8 µm).
10.1186/s12967-016-1040-4 The sequences of PCR primers used for the detection of rat β-actin, β2 microglobulin, GAPDH, TGF-β1, TGF-β type II receptor, IL-6 and its receptor, IL-10 and its type A & B receptors, IL-22 and its type A1 and A2 receptors and MMP9 mRNAs in liver samples including the corresponding genes accession numbers and amplicon sizes.
