# Supplementary figures and images for: The fibrolytic potentials of vitamin D and thymoquinone remedial therapies: insights from liver fibrosis established by CCl4 in rats
Source: J Transl Med. 2016 Sep 29;14:281. doi: 10.1186/s12967-016-1040-4 (PMC5041560; doi:10.1186/s12967-016-1040-4)

## Slide 1
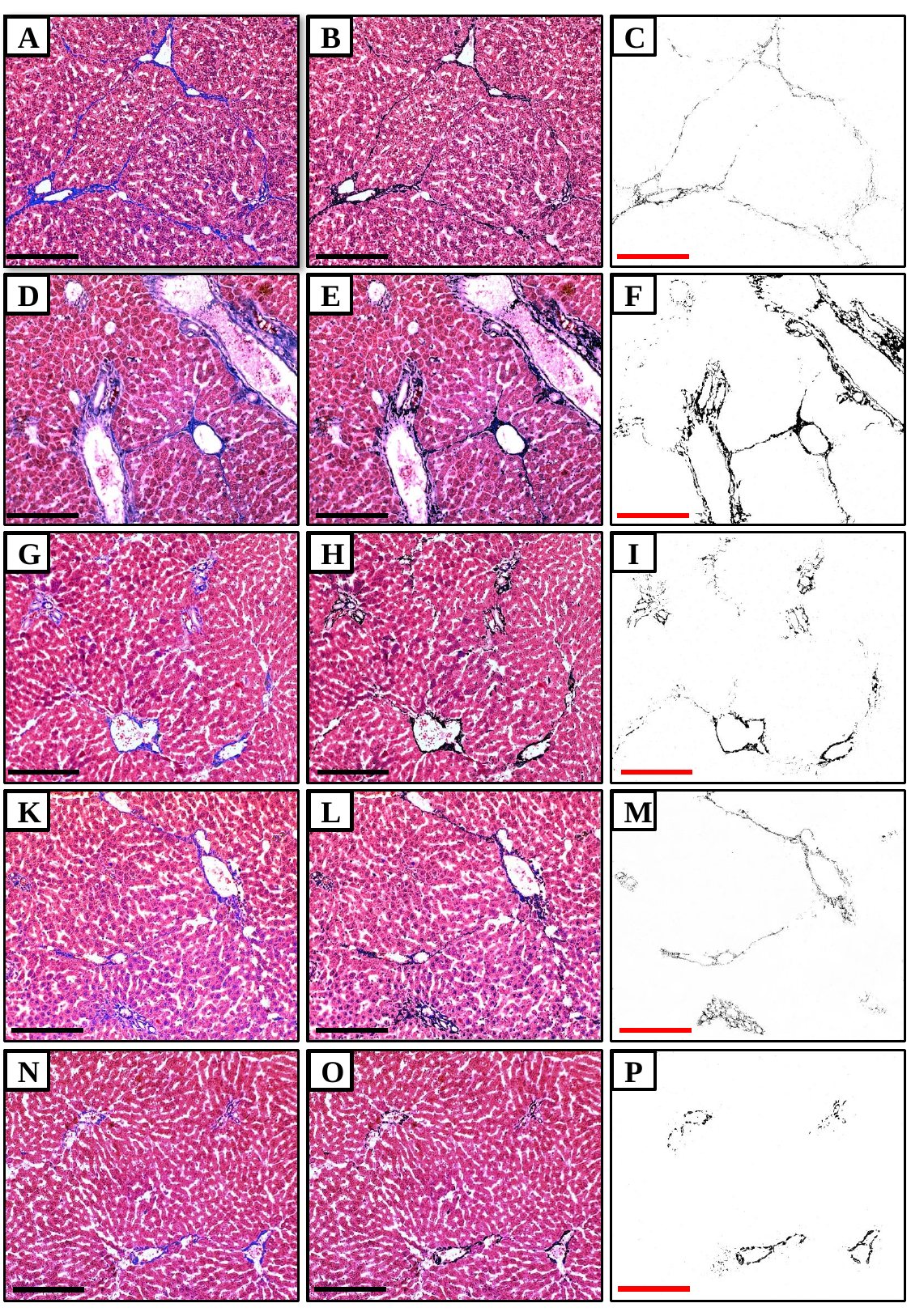

A
B
C
D
E
F
G
H
I
K
L
M
N
O
P

Supplement: Supplementary file 1 — 10.1186/s12967-016-1040-4 Steps of processing the study digital images with ImageJ software for calculating the fibrosis index (% of collagen deposition), which was identified by blue staining, following Masson’s trichrome stain of liver sections from S-PC (1st upper row), L-PC (2nd row), TQ (3rd row), VitD (4th row) and dual therapy (bottom row) groups. The identification and selection of the areas of interest (left column) were done with the guidance of an expert histopathologist. The images were then processed by hue/saturation/brightness (HSB) for color threshold adjustment using ‘black’ as the threshold color to digitally mark and select an area of interest by the software (middle column). This was followed by clearing outside the defined areas of interest to ensure the precision of the identification and selection processes by the software (Right column). All measurements were calculated following calibration with digital photos of corresponding microscopic scale slides captured at the designated magnifications. (×200 magnification, scale bar = 8 µm). [file 12967_2016_1040_MOESM1_ESM.pptx]
