# Supplementary material for: The fibrolytic potentials of vitamin D and thymoquinone remedial therapies: insights from liver fibrosis established by CCl4 in rats
Source: J Transl Med. 2016 Sep 29;14:281. doi: 10.1186/s12967-016-1040-4 (PMC5041560; doi:10.1186/s12967-016-1040-4)
Supplement: Supplementary file 2 — 10.1186/s12967-016-1040-4 The sequences of PCR primers used for the detection of rat β-actin, β2 microglobulin, GAPDH, TGF-β1, TGF-β type II receptor, IL-6 and its receptor, IL-10 and its type A & B receptors, IL-22 and its type A1 and A2 receptors and MMP9 mRNAs in liver samples including the corresponding genes accession numbers and amplicon sizes. [file 12967_2016_1040_MOESM2_ESM.doc]

**Supplementary table 1:** The sequences of PCR primers used for the detection of rat β-actin, β2 microglobulin, GAPDH, TGF-β1, TGF-β type II receptor, IL-6 and its receptor, IL-10 and its type A & B receptors, IL-22 and its type A1 and A2 receptors and MMP9 mRNAs in liver samples including the corresponding genes accession numbers and amplicon sizes.

| **Genes** | **Forward** | **Reverse** | **Amplicon size** |
| --- | --- | --- | --- |
| ***ACTB***  (NCBI: NM_031144.3) | 5’ CGG TCA GGT CAT CAC TAT CG 3’ | 5’ TTC CAT ACC CAG GAA GGA AG 3’ | 79 bp |
| ***B2M***  (NCBI: NM_012512.2) | 5’ TGA AGG AGC CCA AAA CCG TC 3’ | 5’ CCG GAT CTG GAG TTA AAC TGG 3’ | 92 bp |
| ***GAPDH***  (NCBI: NM_017008.4) | 5’ GCA TCT TCT TGT GCA GTG CC 3’ | 5’ GAG AAG GCA GCC CTG GTA AC 3’ | 105 bp |
| ***TGFB1***  (NM_021578.2) | 5’ CCA TGA CAT GAA CCG ACC CT 3’ | 5’ TGC CGT ACA CAG CAG TTC TT 3’ | 141 bp |
| ***TGFBR2***  (NM_031132.3) | 5’AAC AGC GAG ATA CAT GGC CC 3’ | 5’ CAC TCT GGA ACC AAA CGG GG 3’ | 175 bp |
| ***IL6***  (NCBI: NM_012589.2) | 5’ CAC TTC ACA AGT CGG AGG CT 3’ | 5’ TCT GAC AGT GCA TCA TCG CT 3’ | 114 bp |
| ***IL6RA***  (NCBI: NM_017020.3) | 5’ GAG GTC ACA GGC ACT CCT TG 3’ | 5’ TCC GTA CTG ATC CTC GTG GT 3’ | 114 bp |
| ***IL10***  (NCBI: NM_012854.2) | 5’ CCT CTG GAT ACA GCT GCG AC 3’ | 5’ TGG CCT TGT AGA CAC CTT TGT 3’ | 118 bp |
| ***IL10RA***  (NCBI: NM_057193.2) | 5’ ACC ACC GCA TTT ACA GGG TT 3’ | 5’ CCC TTT TGG GAC TTC GAG GG 3’ | 101 bp |
| ***IL10RB***  (NCBI: NM_001107111.1) | 5’ AAA CGT CAC CTT CTG TCC CG 3’ | 5’ GGT CCA TGT CTC AGG CTC AT 3’ | 130 bp |
| ***IL22***  (NCBI: NM_001191988.1) | 5’ TCA GCG GTG ATG ACC AGA AC 3’ | 5’ CCA GTT CCC CGA TCG CTT TA 3’ | 102 bp |
| ***IL22RA1***  (NCBI: NM_001191869.1) | 5’ TGA CTG TGG GAT CCC TGG CTG 3’ | 5’ GTT GCT GGA CTG GAA TTT CAC 3’ | 80 bp |
| ***IL22RA2***  (NCBI: NM_001003404.1) | 5’ CAA CCA GGG AAC TCC CTC AC 3’ | 5’ GGT CCC CCA GCA GTC ATT TT 3’ | 102 bp |
| ***MMP9***  (NCBI: NM_031055.1) | 5’ GAT CCC CAG AGC GTT ACT CG 3’ | 5’ GTT GTG GAA ACT CAC ACG CC 3’ | 132 bp |
